# Research training in an AI world

**DOI:** 10.1172/JCI173283

**Published:** 2023-07-17

**Authors:** Allison R. Chen

**Affiliations:** Cornell University, Ithaca, New York, USA.

Ctrl-C, Ctrl-V, Enter. The cursor blinked briefly and began to auto-generate the following essay.

“Ethical issues can arise in any field, including biomedical engineering and sciences. During my training in biomedical engineering, I encountered an unanticipated ethical issue related to the use of animals in research. While working on a project that involved testing the safety and efficacy of a new medical device, I was confronted with a dilemma about the use of animals in the experimentation process.”

ChatGPT, an artificial intelligence language model, had effortlessly generated 800 words to answer the 2023 Lasker Essay Contest prompt. Consider this. Essay contests teleologically exist to highlight an individual’s writing abilities. Therefore, entering an AI-generated essay for consideration is ethically unacceptable because the submitted essay would reflect the writing ability of the computer program instead of the individual. AI technologies do not challenge the intrinsic purpose of scientific research. Prominent examples of research-AI compatibility, from the prediction of almost all protein structures via Alphafold (1, 2) to the early diagnosis of diseases like Parkinson’s or breast cancer from machine learning–derived markers (3), have advanced our knowledge of biomedical sciences beyond our wildest dreams. ChatGPT and language-learning models (LLMs) may contribute to similar research advancements by increasing the rate of scientific knowledge gathering and communication. As the beneficiaries of this technology, we researchers have a responsibility to anticipate and resolve the unexpected ethical issues of using ChatGPT before harm can occur.

Over 80 percent of scientists, including myself, have used ChatGPT (4) to perform literature reviews, brainstorm experiments, communicate results, or write grants (5).

Some examples of prompts I’ve entered into ChatGPT:

Who is [famous academic], write like [Dr. Seuss/Homer]

Define [unknown term (ex. teleology)] for [research field]

List [grants/fellowships/high impact papers about (ex. LNPs)]

Rewrite [report/presentation] more [concise/academic]

Reply to [email/message] from [colleague/advisor]

Suggest [cell assays/protocol] for [research field (ex. uptake)]

We use it to demystify the work we do, yet the mystery regarding how ChatGPT operates is not truly knowable. Nor can we satisfactorily cite the sources of knowledge it so actively provides. Computer engineers have their terms — algorithms, neural networks, statistical relationships between words, inputs, and outputs — but ultimately, ChatGPT is the newest member of an old club of technologies including zippers, bicycles, microwaves, iPhones; knowledge of how it works isn’t required to use it.

ChatGPT is also part of a more insidious club: technologies that potentially generate and disseminate misinformation (6, 7). A “hallucination,” false or nonsense information presented as fact by an LLM, slips through the guardrails. A high-impact journal publishes convincing fake research-paper abstracts. Certain groups fearing this hypothetical loss of transparency in the scientific process — *Science* magazine, my advisor — have responded by imposing a moratorium on AI-written work until the scientific community reaches a consensus on ethical ChatGPT use. High-level researchers and journals contribute to this dialogue and have suggested that scientists who use ChatGPT should fact-check the generated output and document their use of LLMs in manuscripts and literature searches. Though such suggestions hold merit, as a society we tend to learn less from our successes and more from our mistakes.

Lacking the experience-derived intuition of older researchers, graduate research assistants, I hypothesize, will contribute to more of the mistakes that will shape the ethical guidelines of the future. By the time I began using ChatGPT, I had overcome the “first year PhD” haze of stress and confusion resulting from poorly designed experiments and paper-reading incompetence. I entered a quasi-magical existence where I understood the current field of research. Having combed through lipid nanoparticle literature for over a year, I was humbled to see ChatGPT accurately summarize my thesis project in under a minute. Less impressively, it also hallucinated a list of research articles when I requested citations. Academic advisors and graduate assistants acknowledge that the ability to communicate and evaluate the quality of scientific information is a core skill gained during the PhD experience. In the absence of broader guidance from an older generation of seasoned researchers, my colleagues and I are learning to use ChatGPT through trial and error as we take part in a real-time experiment regarding the ethical use of ChatGPT and its impact on our scientific training.

We have become the case study. By adopting ChatGPT, will my generation develop into tech-savvy researchers wise to the perils of misinformation during a golden age of research or inhabit a more ill-informed world, less capable than our older counterparts?

## Version 1 07/17/2023

Electronic publication

## References

[1] Callaway E (2022). ‘The entire protein universe’: AI predicts shape of nearly every known protein. Nature.

[2] Callaway E (2022). What’s next for AlphaFold and the AI protein-folding revolution. Nature.

[3] https://www.nytimes.com/2019/03/11/well/live/how-artificial-intelligence-could-transform-medicine.html.

[4] https://www.nature.com/articles/d41586-023-00603-2.

[5] https://www.nature.com/articles/d41586-023-00528-w.

[6] [No authors listed] (2023). Tools such as ChatGPT threaten transparent science; here are our ground rules for their use. Nature.

[7] Hutson M (2021). Robo-writers: the rise and risks of language-generating AI. Nature.

